# Supplementary material for: Investigating the Benefit of Combined Androgen Modulation and Hypofractionation in Prostate Cancer
Source: Int J Mol Sci. 2020 Nov 10;21(22):8447. doi: 10.3390/ijms21228447 (PMC7698244; doi:10.3390/ijms21228447)
Supplement: Supplementary file 1 [file ijms-21-08447-s001.zip › Suppl table I.docx]

| **Protein** | **Catalog Number** | **Producer** |
| --- | --- | --- |
| AR | Ab9474 | Abcam |
| PSA | 5365 | Cell Signaling Technology |
| RAD51 | ABE257 | Merck-Millipore |
| Chk2 | 2662 | Cell Signaling Technology |
| p-Chk2 (Thr68) (C13C1) | 2197 | Cell Signaling Technology |
| ATM (D2E2) | 2873 | Cell Signaling Technology |
| Bcl-XL | 2764 | Cell Signaling Technology |
| BAX | 2772 | Cell Signaling Technology |
| HIF-1α (D1S7W) | 36169 | Cell Signaling Technology |
| Vinculin (VLN01) | MA5-11690 | Thermo Scientific |

**Supplementary Table 1.** Complete list of the antibodies used, provided with clones and manufacturer specifications..
