# Supplementary figures and images for: Investigating the Benefit of Combined Androgen Modulation and Hypofractionation in Prostate Cancer
Source: Int J Mol Sci. 2020 Nov 10;21(22):8447. doi: 10.3390/ijms21228447 (PMC7698244; doi:10.3390/ijms21228447)

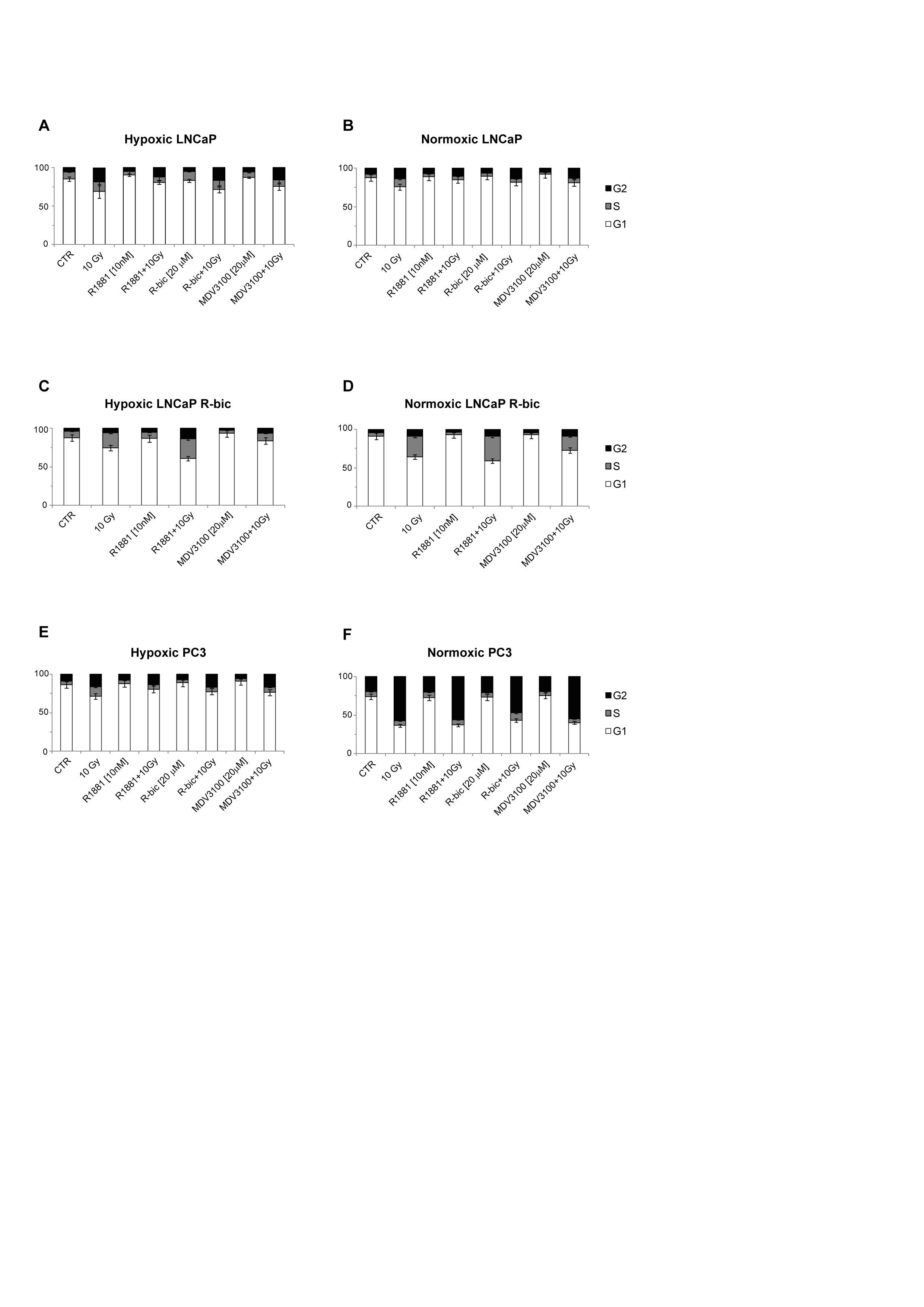

Supplement: Supplementary file 1 [file ijms-21-08447-s001.zip › Suppl figure 3.tif]

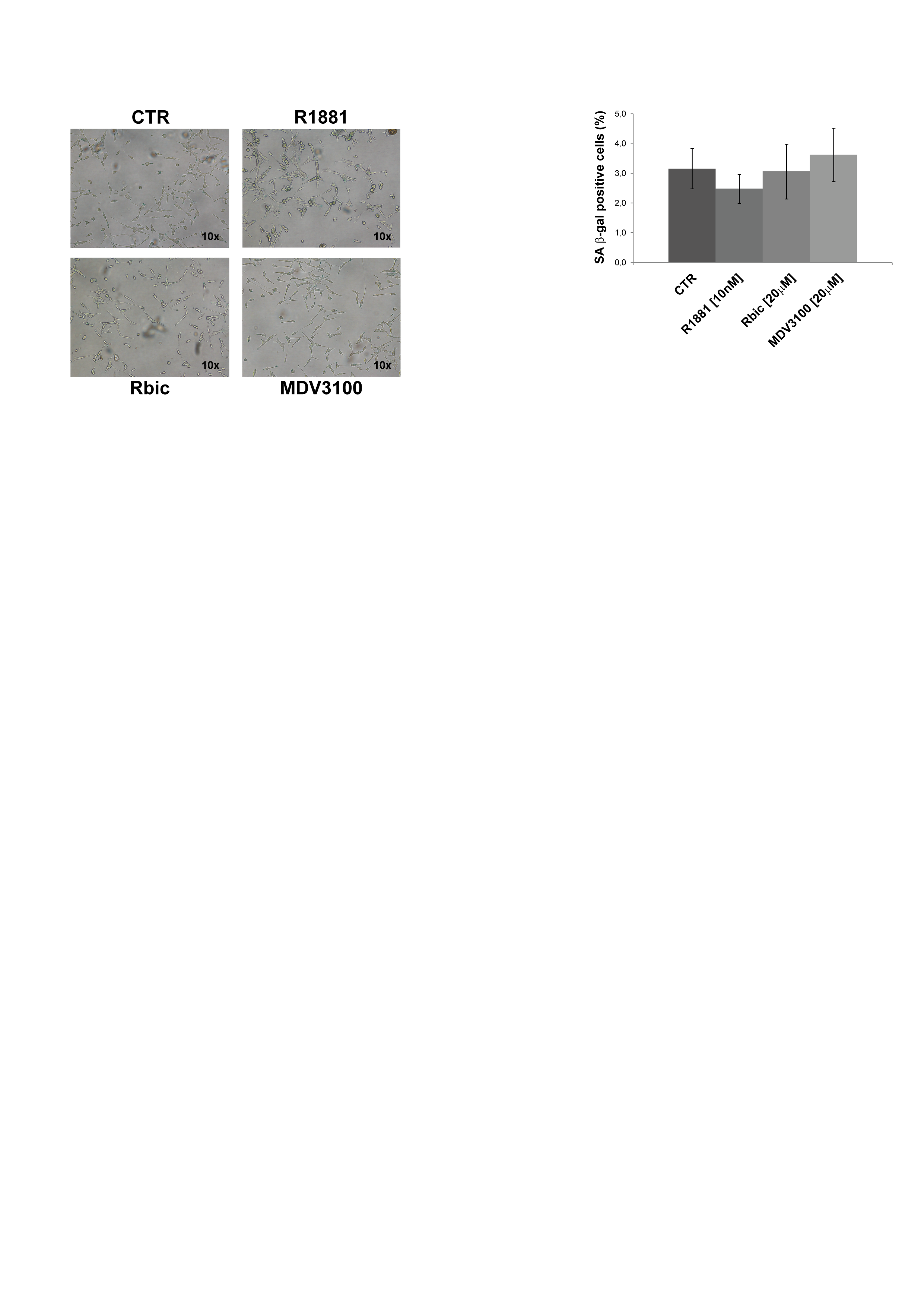

Supplement: Supplementary file 1 [file ijms-21-08447-s001.zip › Suppl figure 4_suppl.tif]

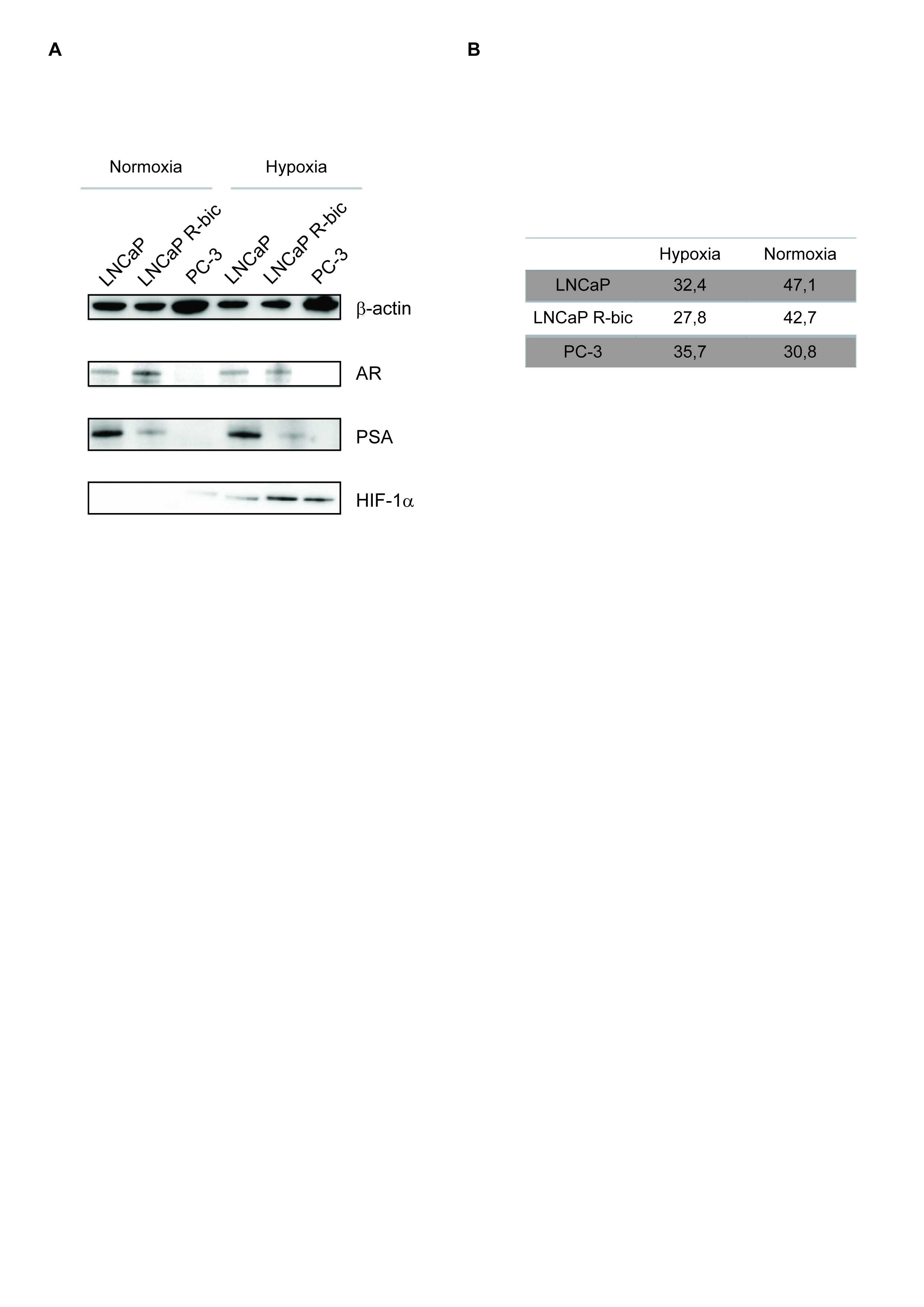

Supplement: Supplementary file 1 [file ijms-21-08447-s001.zip › Suppl figure 1.tif]

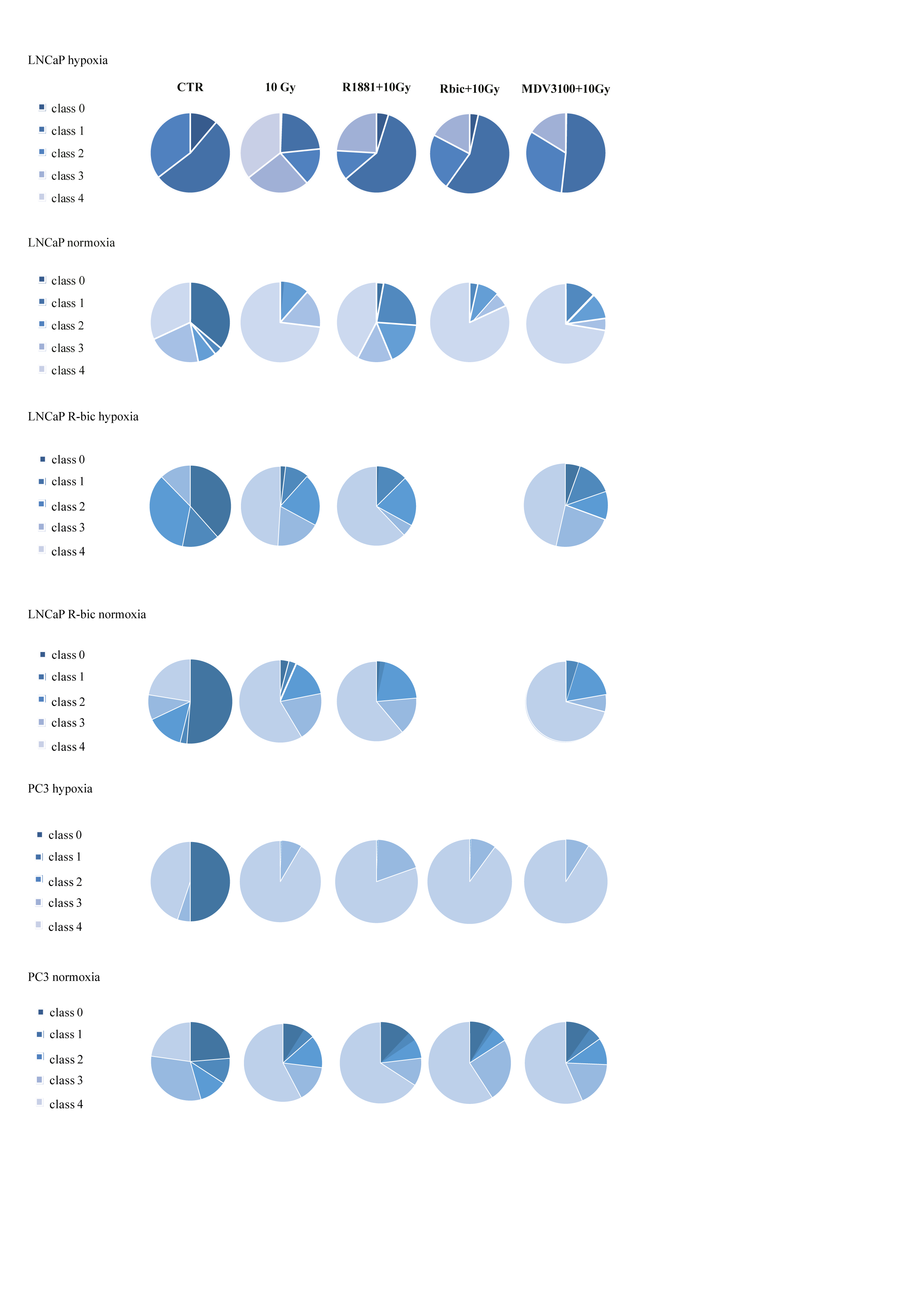

Supplement: Supplementary file 1 [file ijms-21-08447-s001.zip › Suppl figure 2.tif]

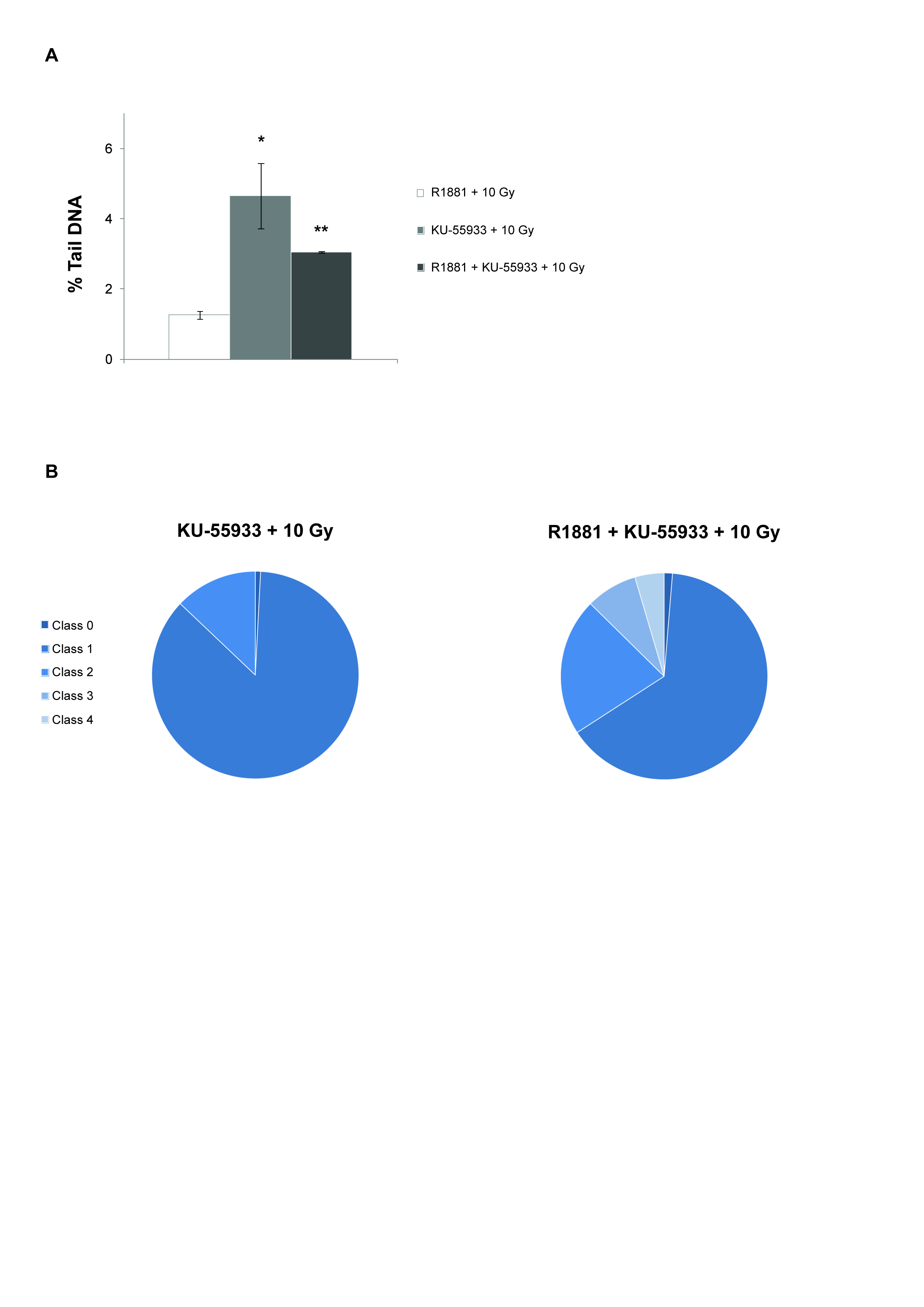

Supplement: Supplementary file 1 [file ijms-21-08447-s001.zip › Suppl figure 5.tif]
